# Transurethral laser ablation using 980-nm diode laser for recurrent non-muscle invasive bladder cancer

**DOI:** 10.37349/etat.2026.1002386

**Published:** 2026-07-28

**Authors:** Hideo Fukuhara, Syo Shimasaki, Erika Yamashita, Yoshitaka Kurano, Kaya Atagi, Ryu Shigehisa, Daigo Takemori, Shinkuro Yamamoto, Hiroto Osakabe, Tomoya Nao, Tsutomu Shimamoto, Satoshi Fukata, Shingo Ashida, Keiji Inoue

**Affiliations:** IRCCS Istituto Romagnolo per lo Studio dei Tumori (IRST) “Dino Amadori”, Italy; Department of Urology, Kochi Medical School, Nankoku 783-8505, Japan

**Keywords:** transurethral laser ablation, transurethral resection of bladder tumor, non-muscle invasive bladder cancer

## Abstract

**Aim:**

Transurethral laser ablation (TULA) for recurrent bladder cancer is a less invasive treatment method for patients. We report here our initial clinical experience with TULA for recurrent bladder cancer.

**Methods:**

This retrospective study analyzed 48 patients. Twenty-four cases were treated with TULA and another 24 cases were treated with transurethral resection of bladder tumor (TURBT) from Feb 2024 to Mar 2025 at Kochi Medical School Hospital. Intraoperative and postoperative outcomes and recurrence-free survival (RFS) were analyzed between TULA and TURBT.

**Results:**

This study showed that TULA using a 980-nm diode laser was significantly shorter than TURBT in terms of perioperative results: operative time, hospital stay, catheterization, bladder irrigation, and postoperative hospital stay. Regarding the occurrence of complications, TULA was observed to be less prevalent than TURBT in all cases. Kaplan-Meier curves showed that TULA was significantly associated with longer RFS compared to TURBT (hazard ratio 4.739; 95% confidence interval, 1.272–17.65; *p* = 0.02039).

**Conclusions:**

Our preliminary results showed that TULA for recurrent bladder cancer is feasible and safe.

## Introduction

In Japan, about 2,200 people are newly diagnosed with bladder cancer each year, and about 9,000 people die. The number of new cases of bladder cancer is increasing every year [1]. More than 50% of non-muscle invasive bladder cancer (NMIBC) patients experience recurrence within one to two years after transurethral resection of bladder tumor (TURBT) surgery, and NMIBC is characterized by a very high postoperative recurrence rate [2]. Furthermore, TURBT is an invasive surgical procedure with many intra- and postoperative complications such as bleeding, bladder perforation, and obturator nerve reflex. The risk of recurrence of bladder cancer will increase with the elderly population due to the increase in life expectancy. Therefore, especially in elderly and frail patients with NMIBC, repeated recurrences may require multiple hospitalizations for TURBT. This leads to a huge emotional and financial stress for the patient. If bladder cancer recurs frequently, the patient is at risk of developing muscle invasive bladder cancer and will require radical cystectomy. Therefore, it is desirable to create a new treatment method that is less burdenful to patients and reduces the risk of postoperative recurrence of NMIBC or progression to muscle invasive cancer compared to conventional TURBT in bladder cancer treatment.

Treatment of large or multiple bladder tumors with TURBT under anesthesia has advantages in terms of cancer control with complete resection. However, if the recurrent tumor is a small low-risk bladder tumor, TURBT under anesthesia may result in a shorter operative time and a higher risk of anesthesia and complications. Therefore, TURBT without spinal or general anesthesia is not feasible due to its painful and invasive procedure for patients.

Treatment with 980-nm wavelength diode lasers has been applied to evaporation techniques due to the property that its energy is absorbed by both water molecules and oxidized hemoglobin. Diode lasers at 980 nm have been reported to provide excellent hemostasis and evaporation in contact laser vaporization of the prostate (CVP) surgery for benign prostatic hyperplasia (BPH) in the urological field [3–5]. This also allows CVP surgery to have a high hemostatic performance and the advantage of being performed without discontinuing anticoagulant medications. Naturally, the 980 nm diode laser evaporation technique can be applied to NMIBC. Hermann et al. [6, 7] reported that transurethral laser ablation (TULA) for NMIBC is superior to conventional TURBT in terms of lower complication rates and recurrence rates at 12 months after surgery. TULA using a combination of a laser fiber with a flexible cystoscope can reduce the stress on the urethra and significantly decrease pain. This point leads to the practice of treatment under local anesthesia without the need for spinal or general anesthesia.

There are no reports from Japan on the efficacy and safety of TULA for NMIBC. Therefore, this study reports initial experience of treatment efficacy regarding intraoperative and postoperative outcomes of TULA for recurrent NMIBC in Japan.

## Materials and methods

### Patients’ characteristics

NMIBC treated with TURBT or TULA from February 2024 to March 2025 at Kochi Medical School were included in this analysis. TULA was performed for recurrence of Ta bladder tumor, and no more than 2 cm. In addition, the attending physician made a comprehensive assessment and determined that TULA was appropriate. TURBT was performed for all NMIBC patients other than those eligible for TULA. These cases were conducted over a series of consecutive time periods, and a retrospective analysis was performed. The Institutional Review Board of Kochi University Medical School Hospital approved this study (approval no. 2024-85) and waived the need for informed consent. Data were obtained from all patients who completed a general consent form based on the opt-out policy at Kochi Medical School Hospital. This study was conducted in accordance with ethical principles for medical research on human subjects, including research on identifiable human materials and data, stated in the Declaration of Helsinki (the 64th World Medical Association General Assembly, Fortaleza, Brazil, October 2013). Patients’ demographic data, surgical parameters, and pathological results were obtained from our database. The baseline characteristics included age, gender, smoking, multiplicity of tumor, tumor diameter, pathological T stage, tumor grade, etc. The intraoperative and postoperative parameters were total operation time, duration of catheterization, total length of hospitalization, length of postoperative hospitalization, intraoperative complication of bladder perforation, hematuria after operation, liver dysfunction, and postoperative febrile urinary infection.

### Perioperative procedure

The objective of this study was to investigate the safety and feasibility of 980-nm wavelength diode laser ablation for recurrent bladder cancer of recurrent Ta NMIBC. A detailed history and physical examination, blood tests, urinalysis, serum electrolytes, blood coagulation tests, and computed tomography (CT) and magnetic resonance imaging (MRI) were performed for all patients preoperatively. Prior to TULA, an outpatient cystoscopy was performed to identify the location, size, and number of tumors. Subsequently, all patients underwent TULA or TURBT in an inpatient setting. The occurrence of intraoperative and postoperative complications was monitored for a period of 30 days.

### Surgical techniques of TURBT and TULA

**Figure 1:**
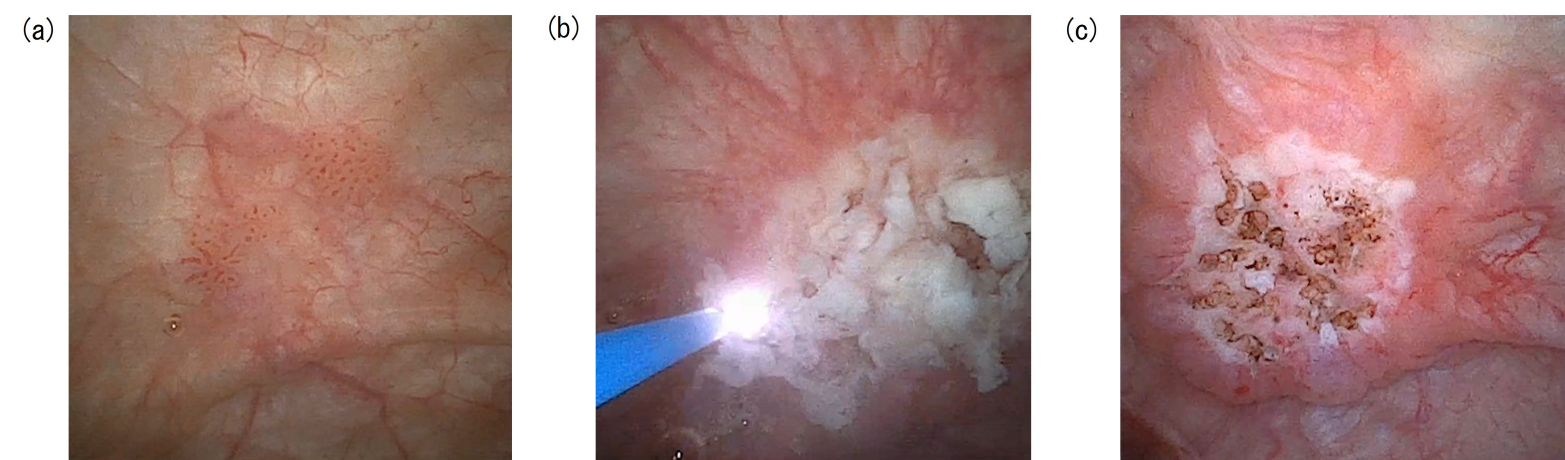
**Cystoscopy findings.** (**a**) Recurrent bladder tumors seen on outpatient follow up. (**b**) White degenerated tumor tissue during laser ablation. (**c**) Mucosal lesions after laser ablation.

TURBT under general/spinal anesthesia was performed by a urologist using Karl Storz (Tuttlingen, Germany) equipment with bipolar resection using white light cystoscopy. The use of photodynamic diagnosis (PDD) was based on the judgment of each physician. Postoperatively, a single dose of epirubicin dissolved in 40 mL saline was injected into the bladder according to Japanese Urological Association (JUA) guidelines. Postoperative balloon indwelling and bladder irrigation were performed. TULA was performed in the lithotomy position under either general/spinal anesthesia or local anesthesia without obturator nerve block. General/spinal anesthesia cases were performed using a 23Fr continuous perfusion rigid scope (KARL STORZ SE & Co.) for TULA. For local anesthesia cases, a 16.2Fr single-use flexible cystoscope (Claritoron system, Scivita Medical Technology Co., Ltd.) was used for TULA. The irrigation fluid was composed of normal saline at room temperature. Initially, the tumor tissue for biopsy was collected using biopsy forceps. Subsequently, laser irradiation was performed on the tumor tissue. The laser irradiation was conducted utilizing a 980-nm wavelength diode laser generator (LEONARDO 180, biolitec® Holding GmbH & Co KG) with a green 532-nm guide light and a forward direct-radiating 400 µm TULA laser fiber. The application of laser irradiation was performed through the utilization of the ‘painting technique’, which entailed the gentle stroking of the tissue surface. The laser output power was set at 5 W or 8 W with pulsed wave mode (0.1 s irradiation, 0.1 s interval) or continuous mode. The distance between the fiber tip and the target tissue was 0 mm, at which the fiber touched the bladder wall without pressure, or 2 mm until all tumor tissue was white-degenerated and coagulated (Figure 1). After laser irradiation, deep tumor tissue in the laser-treated areas was collected using cup biopsy forceps in only the first four cases to measure the laser penetration depth based on the presence of viable cancer cells and tissue degeneration. A single postoperative epirubicin dose was not administered. Postoperatively, balloon indwelling was standardized for general/spinal anesthesia cases, while its use was omitted for local anesthesia cases. Postoperative bladder irrigation was performed based on each physician’s judgment.

### Evaluation of TULA treatment efficacy and adverse events (AEs)

Following TURBT or TULA, cystoscopy and urine cytology were performed on an outpatient basis at three-month intervals. The outpatient follow-up protocol was as follows: Cystoscopy and urine cytology were performed every three months. Recurrence was defined as intravesical tumor recurrence that was pathologically confirmed as urothelial carcinoma. A systematic patient chart review and post-procedure outpatient information were used to document any side effects and complications.

### Statistical analysis

To examine significant differences in the distribution of categorical variables among two groups, Fisher’s exact test for non-parametric data and Student’s *t*-test for parametric data were performed on the overall contingency table. Kaplan-Meier analysis was used to visualize and evaluate differences in recurrence-free survival (RFS). Multivariable Cox regression model was used to evaluate the association of RFS with each treatment outcome. Statistical analysis was performed using EZR, a graphical user interface for R. All *p*-values of *p* < 0.05 indicated statistical significance.

## Results

### Intraoperative and postoperative outcomes

The total number of cases in this study was 48, of which 24 were TURBT and 24 were TULA (Table 1).

**Table 1:** Patients’ characteristics between TULA and TURBT.

**Category**	**Total**	**TULA**	**TURBT**	*p*-value *
Characteristics	*n* = 48	*n* = 24	*n* = 24	-
Sex, *n* (%)				> 0.999
Male	39	20 (83)	19 (79)	-
Female	9	4 (17)	5 (21)	-
Age, median (IQR)	76 (72–86.25)	76 (71.75–84.75)	76.5 (73.5–80.75)	-
Smoking, *n* (%)				0.223
Current	11	8 (33)	3 (13)	-
Former	25	10 (42)	15 (63)	-
Never	12	6 (25)	6 (25)	-
ECOG-performance status, *n* (%)				0.096
0	22	11 (46)	11 (46)	-
1	21	8 (33)	13 (54)	-
2	2	2 (8)	0 (0)	-
3	3	3 (13)	0 (0)	-
Use of antithrombotic agents (temporarily discontinued perioperatively), *n* (%)				0.359
Yes	16	10 (42)	6 (25)	-
No	32	14 (58)	18 (75)	-
History of BCG therapy, *n* (%)				1
Yes	4	2 (8)	2 (8)	-
No	44	22 (92)	22 (92)	-
Number of prior TURBTs, *n* (%)				< 0.001
0	10	0 (0)	10 (42)	-
1	16	7 (29)	9 (38)	-
2	10	8 (33)	2 (8)	-
≥ 3	12	9 (38)	3 (13)	-
Preoperative urine cytology (max), *n* (%)				0.192
Negative	36	21 (88)	15 (63)	-
Atypical/Suspicious	5	2 (8)	3 (13)	-
Positive	5	1 (4)	4 (17)	-
N/A	2	0 (0)	2 (8)	-
Pathological T stage, *n* (%)				0.033
Ta	43	24 (100)	19 (79)	-
T1	5	0 (0)	5 (21)	-
Pathological tumor grade, *n* (%)				0.009
Low-grade	17	12 (50)	5 (21)	-
High-grade	25	12 (50)	13 (54)	-
unknown	6	0 (0)	6 (25)	-
Tumor number, *n* (%)				0.148
Solitary	24	15 (63)	9 (38)	-
Multiple	24	9 (38)	15 (63)	-
Tumor size (mm), *n* (%)				0.093
≤ 30	38	20 (83)	18 (75)	-
> 30	4	0 (0)	4 (17)	-
unknown	6	4 (17)	2 (8)	-
Main tumor location, *n* (%)				0.263
Trigone	5	1 (4)	4 (17)	-
Right lateral wall	10	4 (17)	6 (25)	-
Left lateral wall	5	4 (17)	1 (4)	-
Anterior wall	4	4 (17)	0 (0)	-
Posterior wall	14	7 (29)	7 (29)	-
Dome	2	1 (4)	1 (4)	-
Bladder neck (internal urethral orifice)	4	1 (4)	3 (13)	-
urethra	4	2 (8)	2 (8)	-
Use of PDD, *n* (%)				< 0.001
Yes	15	1 (4)	14 (58)	-
No	33	23 (96)	10 (42)	-

*: Fisher’s exact test.

The TURBT group included 21 cases of general anesthesia and 3 cases of spinal anesthesia. The TULA group included 12 cases of general anesthesia, 2 cases of spinal anesthesia, and 10 cases of urethral anesthesia. The mean total operation time was 39.04 ± 19.03 minutes in the TULA group and 60.21 ± 39.79 minutes in the TURBT group (*p* < 0.05). The mean duration of bladder irrigation was 0.33 ± 0.76 days in the TULA group and 1.92 ± 0.28 days in the TURBT group (*p* < 0.05). The mean duration of catheterization was 1.17 ± 1.00 days in the TULA group and 2.17 ± 0.76 days in the TURBT group (*p* < 0.05). The mean length of hospitalization was 5.96 ± 1.81 days in the TULA group and 7.46 ± 1.47 days in the TURBT group (*p* < 0.05). The mean length of postoperative hospitalization was 2.13 ± 1.7 days in the TULA group and 3.83 ± 1.34 days in the TURBT group (*p* < 0.05) (Table 2).

**Table 2:** Comparison of perioperative outcomes between TULA and TURBT.

**Perioperative outcomes**	**TULA (*n* = 24)**	**TURBT (*n* = 24)**	*p*-value *
Operating time (min)	39.04 ± 19.03 (13–91)	60.21 ± 39.79 (17–199)	*p* < 0.05
Bladder irrigation (day)	0.33 ± 0.76 (0–2)	1.92 ± 0.28 (1–2)	*p* < 0.05
Catheterization (day)	1.17 ± 1.00 (0–2)	2.17 ± 0.76 (0–4)	*p* < 0.05
Hospital stay (day)	5.96 ± 1.81 (4–13)	7.46 ± 1.47 (5–11)	*p* < 0.05
Hospital stay after operation (day)	2.13 ± 1.70 (1–9)	3.83 ± 1.34 (2–6)	*p* < 0.05

*: *t*-test.

In the postoperative period, there were no patients that received either immediate single pirarubicin instillation or intravesical bacille Calmette-Guerin (BCG) therapy for TULA cohort. Seventeen patients received immediate single pirarubicin instillation and four patients received intravesical BCG therapy for TURBT cohort.

Intraoperative and postoperative complications in the TURBT group included dysuria: 2 cases (8.3%), hematuria after operation: 1 case (4.2%), liver dysfunction: 1 case (4.2%), urinary tract infection: 1 case (4.2%), and bladder perforation: 1 case (4.2%). Dysuria, urinary tract infection, and bladder perforation were all classified as Clavien-Dindo classification grade I. No AEs were observed in the TULA group (Table 3). No concerns were observed in the TULA group beyond the 30-day postoperative follow-up period.

**Table 3:** Comparison of intraoperative and postoperative adverse events between TULA and TURBT.

**Perioperative adverse events**	**TRUBT, *n* (%)**	**TULA, *n* (%)**
Dysuria	2 (8.3)	0
Hematuria after operation	1 (4.2)	0
Liver dysfunction	1 (4.2)	0
Urinary tract infection	1 (4.2)	0
Bladder perforation	1 (4.2)	0

**Figure 2:**
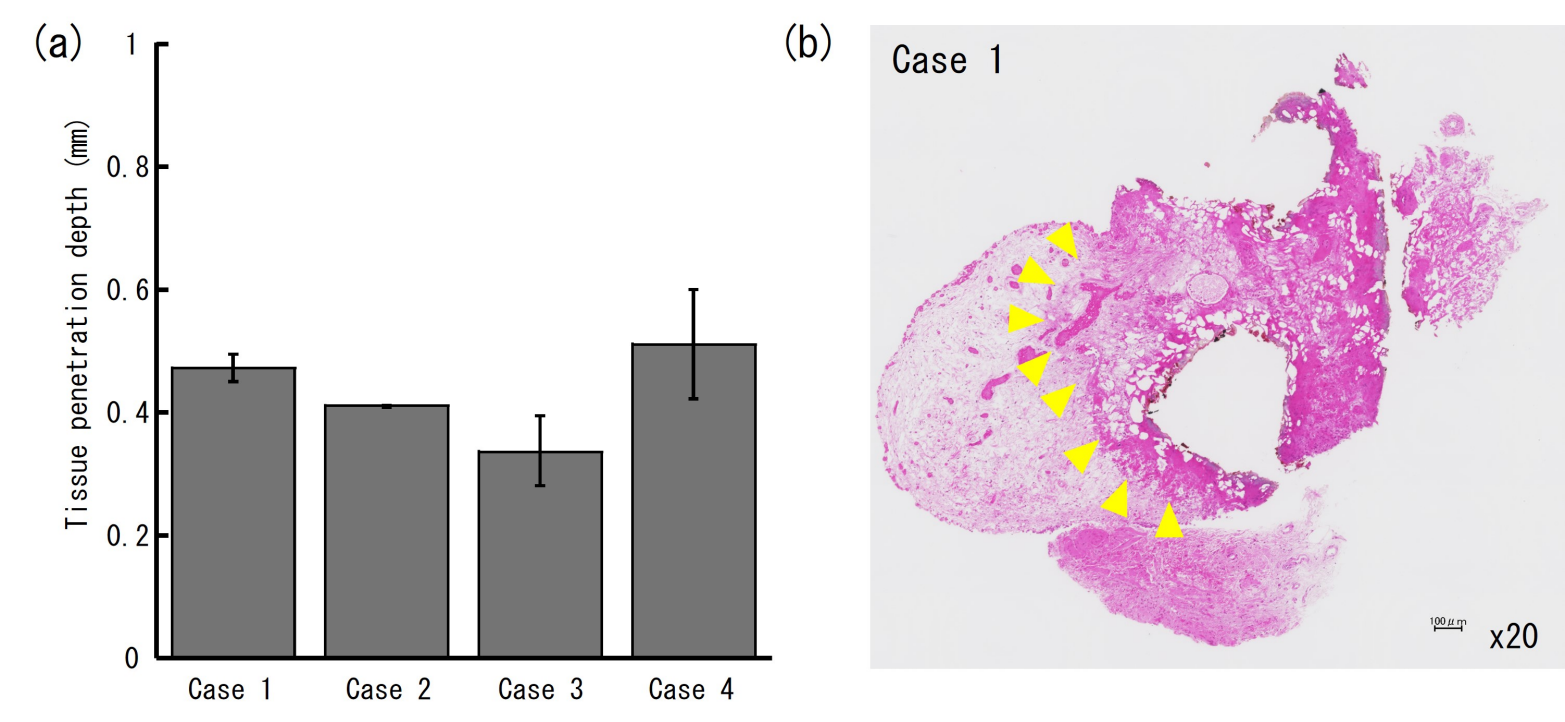
**Analysis of the depth of tissue degeneration.** (**a**) Tissue degeneration depth of biopsy specimens in human bladder tumor base in four cases. (**b**) Histological findings in human bladder tissue degeneration by laser irradiation (HE staining, case 1). Yellow arrow: tissue degeneration by laser irradiation can be observed in specimens from deep biopsies of the bladder tumor base.

No histologically confirmed viable cancer cells were identified in four cases. The average depth of tissue degeneration in biopsy specimens of bladder tumor tissue base from laser-treated area was 0.43 ± 0.08 mm on HE staining in the four patients with TULA (Figure 2a). The maximum depth of tissue degeneration did not reach 0.5 mm in any of the four cases (Figure 2b).

### RFS of TULA for recurrent NMIBC

**Figure 3:**
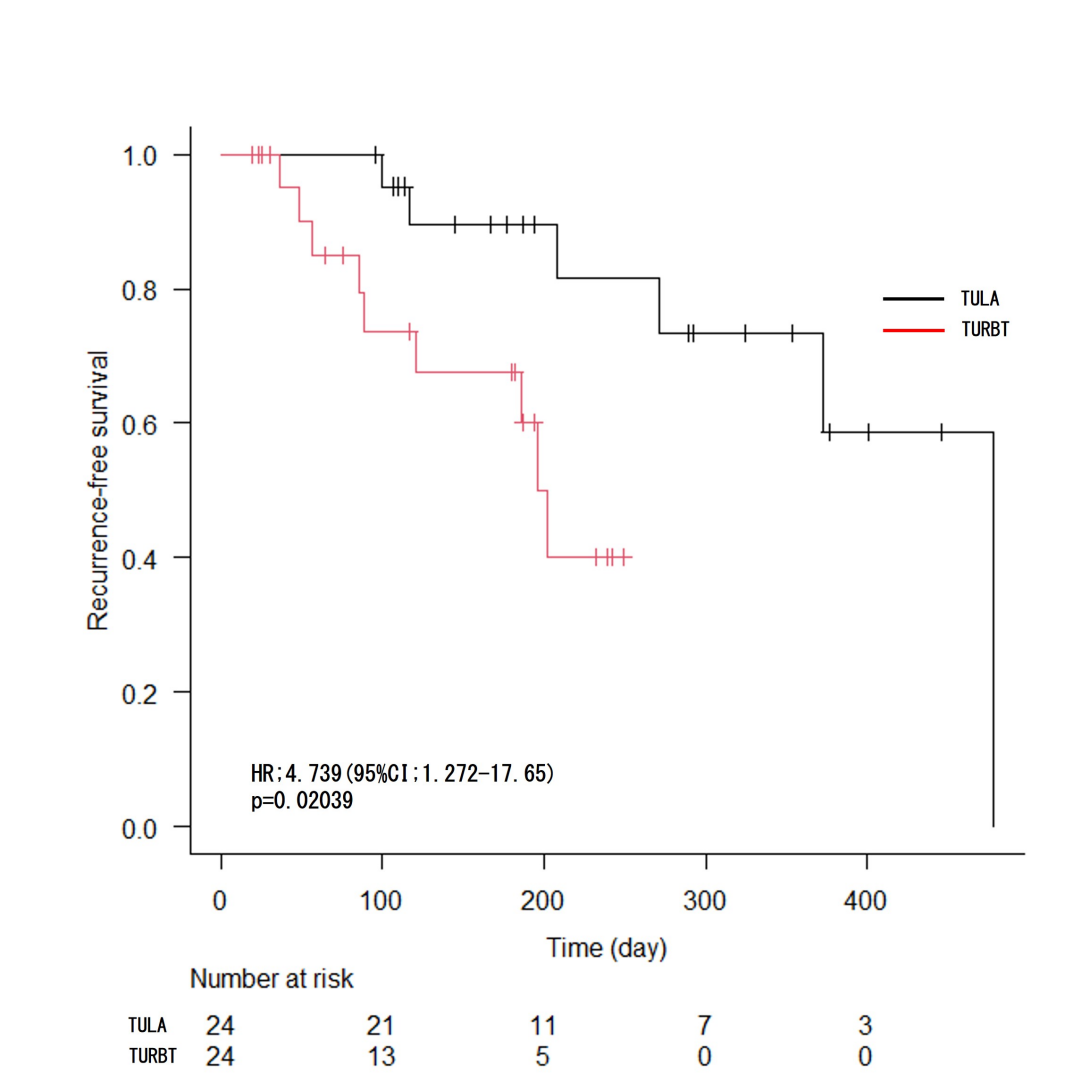
**Comparison of recurrence-free survival between TULA and TURBT. Kaplan-Meier analysis for recurrence-free survival between TULA and TURBT.** Vertical marks on plots represent censored events.

The present study investigates the effect of TULA treatment by examining the postoperative RFS. During a median follow-up period of 6.45 months, Kaplan-Meier curves estimated that TULA was significantly associated with longer RFS compared to TURBT (hazard ratio 4.739; 95% confidence interval, 1.272–17.65; *p* = 0.02039) (Figure 3). TULA demonstrated superiority to TURBT in terms of recurrence rate.

## Discussion

TURBT is the standard and essential surgical technique for NMIBC. The skill of the surgeon has a significant impact on outcomes and complication rates in TURBT. Intraoperative complications of TURBT include rebleeding, bladder perforation, obturator nerve reflex, and urinary tract infection. To reduce the incidence of such complications by TURBT, Li et al. [8] were the first to investigate evaporation using several laser generators for NMIBC. They reported that ablation with the holmium- yttrium-aluminum-garnet (YAG) laser, 2,000-nm and 1,900-nm thulium-YAG lasers, and potassium titanyl phosphate (KTP) green light laser not only reduced the complication rate but also decreased the postoperative recurrence rate compared to TURBT [8–13]. Their review revealed that laser ablation for NMIBC may be superior to conventional TURBT.

The 980-nm wavelength diode laser technology is expected to bring many advantages to the surgical treatment of NMIBC. The 980-nm wavelength characteristic allows efficient non-contact hemostasis and high hemostatic performance [14, 15]. First, the 980-nm wavelength diode laser has a large absorption coefficient for oxidizing hemoglobin to heat and reliably coagulate the blood vessels of the tumor and interrupt blood flow. Secondly, the low absorption coefficient of water molecules allows penetration into deep tissues and ensures that tumor cells at the base of the tumor are also degenerated. This reduces the possibility of residual tumor cells due to incomplete ablation and allows for a highly curative therapeutic effect. Mao et al. [16] reported a study of 980-nm diode laser en-block resection in 40 NMIBC patients. They reported that 980-nm diode laser can deliver a good hemostatic effect on vascular hemorrhage by applying non-contact laser irradiation from the bleeding point to the tissue surface. Laser thermal transfer to the tissue allows precise incisions with minimal bleeding. As a result, the length of bladder irrigation could be markedly shortened, and no posterior hemorrhage was observed.

European Association of Urology (EAU) guidelines recommend the use of diode lasers for the treatment of bladder outlet obstruction (BOO) and BPH. Laser ablation can provide complete control of intraoperative bleeding in patients on anticoagulants or hemorrhagic disease. Furthermore, laser ablation is listed in the office-fulguration section under evidence level 1a as laser photocoagulation for recurrent Ta low-grade NMIBC [17]. It is stated that laser ablation for carefully selected recurrent NMIBC on an outpatient setting offers important economic and institutional organizational savings and reduces the burden and annoyance of hospitalization for patients and their family.

Pedersen et al. [18] reported a randomized controlled trial (RCT) of recurrent low-grade Ta NMIBC (intermediate risk) treated with laser ablation using the 980-nm diode laser in an outpatient clinic under local anesthesia. They compared TURBT under general anesthesia with TULA under local anesthesia for RFS at 4 months postoperatively. The 4-month postoperative RFS rate was 8% higher for TULA, postoperative quality of life was better with TULA, and minor complications were less frequent with TULA. Thus, TULA is a less invasive procedure with the same oncological outcome and improved postoperative quality of life compared to TURBT. Furthermore, repeated TURBT under general anesthesia has been reported to increase non-cancer specific mortality in low-risk Ta NMIBC patients. There is potential for a lower perioperative complication rate and lower non-cancer specific mortality by replacing TURBT with TULA.

This study showed TULA using a 980-nm diode laser was significantly shorter than TURBT in terms of perioperative results: operative time, hospital stay, catheterization, bladder irrigation, and postoperative hospital stay. Regarding the occurrence of complications, TULA was observed to be less prevalent than TURBT in all cases. The findings suggest that TULA may constitute a less invasive treatment compared with TURBT in NMIBC. Furthermore, postoperative RFS was found to be significantly prolonged compared to TURBT. This study demonstrated favorable outcomes with respect to postoperative recurrence in TULA. Elderly patients with NMIBC with multiple diseases require gentler treatment than conventional TURBT under general anesthesia. The utilization of the diode laser technique has the potential to facilitate this objective. In addition, this diode laser technique has the potential to provide painless treatment for the majority of low-grade urothelial carcinomas using a flexible cystoscope under local anesthesia in an outpatient setting.

From a clinical perspective, it is important to consider some limitations of this study. Firstly, the study is a single-center, preliminary, retrospective study, and the follow-up period is short due to the small number of eligible patients and the low external validity of our results. Furthermore, the results of TULA treated by a single surgeon and the method of enrolling patients may have introduced selection bias and affected the validity of the conclusions. Secondly, there may be potential bias in the patient backgrounds of the TULA and TURBT cohorts. This study was a retrospective observational study. Since treatment selection was left to the discretion of the attending physician, differences in background factors occurred between groups. Furthermore, due to the nature of retrospective studies and constraints related to the number of cases and missing data, it was difficult to perform adequate statistical adjustments using propensity score analysis or multivariate analysis. This study was unable to clearly demonstrate that TULA is superior to TURBT regarding RFS. The RFS results were exploratory and not definitive. Consequently, the necessity for large-scale prospective case studies incorporating multicenter studies and protracted follow-up periods is apparent in order to resolve these limitations in the future.

In this study, the results of using a diode laser at 980-nm wavelength for NMIBC patients proved to be a promising, effective, and safe treatment. These laser techniques have demonstrated excellent hemostatic properties and satisfactory postoperative results. This study provides a basis for further research on the utilization of diode laser in ablation techniques in future studies. Long-term follow-up incorporating prospective RCTs is needed to validate our promising short-term results.

## Abbreviations

AEs: adverse events
BCG: bacille Calmette-Guerin
BOO: bladder outlet obstruction
BPH: benign prostatic hyperplasia
CT: computed tomography
CVP: contact laser vaporization of the prostate
EAU: European Association of Urology
JUA: Japanese Urological Association
KTP: potassium titanyl phosphate
MRI: magnetic resonance imaging
NMIBC: non-muscle invasive bladder cancer
PDD: photodynamic diagnosis
RCT: randomized controlled trial
RFS: recurrence-free survival
TULA: transurethral laser ablation
TURBT: transurethral resection of bladder tumor
YAG: yttrium-aluminum-garnet

## Declarations

### Availability of data and materials

The raw data supporting the conclusions of this manuscript will be made available by the authors to any qualified researcher.

### Copyright

© The Author(s) 2026.
